# The Association between Mercury and Lead Exposure and Liver and Kidney Function in Pregnant Surinamese Women Enrolled in the Caribbean Consortium for Research in Environmental and Occupational Health (CCREOH) Environmental Epidemiologic Cohort Study

**DOI:** 10.3390/toxics10100584

**Published:** 2022-10-04

**Authors:** Sheila A. R. Kort, Jeffrey Wickliffe, Arti Shankar, Martin Shafer, Ashna D. Hindori-Mohangoo, Hannah H. Covert, Maureen Lichtveld, Wilco Zijlmans

**Affiliations:** 1 Faculty of Medical Sciences, Anton de Kom University of Suriname, Paramaribo, Suriname; 2 Department of Environmental Health Sciences, School of Public Health, University of Alabama at Birmingham, Birmingham, AL 35294, USA; 3 Department of Biostatistics and Data Science, School of Public Health and Tropical Medicine, Tulane University, New Orleans, LA 70112, USA; 4 Wisconsin State Laboratory of Hygiene, University of Wisconsin-Madison, Madison, WI 53718, USA; 5 Foundation for Perinatal Interventions and Research in Suriname (Perisur), Paramaribo, Suriname; 6 School of Public Health and Tropical Medicine, Tulane University, New Orleans, LA 70112, USA; 7 Department of Environmental and Occupational Health, School of Public Health, University of Pittsburgh, Pittsburgh, PA 15261, USA

**Keywords:** heavy metals, pregnant women, liver function, kidney function, urban, rural, interior

## Abstract

Exposure to mercury (Hg) and lead (Pb) may have an effect on pregnant women. We assessed the effect of exposure to mercury and lead on liver and kidney functions in a subcohort of pregnant women who participated in the Caribbean Consortium for Research in Environmental and Occupational Health (CCREOH)—Meki Tamara, study. From 400 women aged 16–46 living in rural, urban, and interior regions of Suriname, we measured blood mercury and blood lead levels. Creatinine, urea, and cystatin C were measured to assess kidney function, and aspartate amino transferase (AST), alanine amino transferase (ALT), and gamma-glutamyl transferase (GGT) were measured to assess liver function. Education, region, and ethnicity showed significant differences for both blood mercury and lead levels, which all had *p*-values < 0.001. Creatinine and urea were elevated with higher mercury blood levels. Our findings also suggest a relationship between high mercury blood levels and potential harmful effects on liver and kidney function.

## 1. Introduction

Exposure to toxic metals, such as mercury and lead, can be of special concern during pregnancy both for the mother and the unborn child. These metals may induce kidney and liver disease, which may result in adverse birth outcomes such as a low Apgar score or preterm birth [1,2]. Exposure to mercury may lead to kidney disease [3], with the proximal tubule being the main site for kidney damage [4]. The prevalence of kidney impairment in pregnant women is estimated to be between 0.6 and 3% [5,6]. Organic mercury is less toxic to the kidneys than inorganic mercury [4], and it is mostly inorganic mercury that is taken up by the kidneys [7]. Mercury has an affinity for sulfhydryl groups, also called thiols, which facilitate an interaction with several molecules such as albumin, glutathione, and cysteine. Mercury can contaminate multiple environmental media, including air, water, and soil, and can enter the body through food consumption or absorption through the skin. Exposure sources of mercury, both organic and inorganic forms, to humans include artisanal and small-scale goldmining (ASGM), dental amalgam, contaminated water, and fish consumption [8,9,10,11,12,13].

Lead, another heavy metal of concern to pregnant women, may also affect kidney function. Environmental lead exposure is associated with a decrease in creatinine clearance and as a result may lead to impaired renal function [14]. Lead can enter the body through ingestion or inhalation, and, in rare cases, through dermal absorption. Once in the body, it is transported by blood to the soft tissues, organs, and bone. Adverse health effects that have been associated with lead exposure include systemic effects (anemia, hearing loss) and effects on the nervous system and reproductive system [15]. Lead has a wide variety of uses, including as an alloy, for example in pipes, batteries, radiation protection shields, paints, dyes, cosmetics, and formerly in gasoline. Consumption of contaminated manioc and game is also considered a source of lead exposure in French Guyana and Canada, respectively [16,17].

Previous studies of lead exposure and liver function tests showed a statistically significant correlation between alanine amino transferase (ALT) and lead exposure [18,19]. The influence of environmental lead on AST, ALT, and GGT has been indicated in adult women [20].

When lead enters the body, it is transported by blood to the soft tissues, bone, and organs. Most of the lead in soft tissue is in the liver. Organic forms of lead are metabolized by Cytochrome P450, a complex enzyme in the liver. This same enzyme is responsible for metabolizing vitamin D to 1,25 dihydroxyvitamin D in the liver, giving lead the opportunity to interfere with this metabolism and causing a systemic effect. 

Most studies have placed focus on birth outcomes rather than on the deleterious effect these metals may have on pregnant women. During pregnancy, liver and kidney function are altered and may deteriorate, resulting in clinical conditions such as hyperemesis gravidarum, preeclampsia, and acute fatty liver disease [21]. Additionally, slight changes in liver function tests may occur in the third trimester, but overall reference ranges remain stable and within normal limits throughout pregnancy [22,23]. The reference ranges of the liver enzymes aspartate amino transferase (AST), alanine amino transferase (ALT), and gamma-glutamyl transferase (GGT) are 7–45 U/L, 5–35 U/L and 8–36 U/L, respectively [24]. Elevated levels of these enzymes may indicate damaged liver tissue cells.

Both creatinine and urea are considered to reflect the Glomerular Filtration Rate (GFR).

Creatinine is one of the end products of muscle catabolism and is secreted unchanged through glomerular filtration. Urea is a primary metabolite that is derived from dietary proteins. Urea on its own has low clinical significance because it is not a very sensitive test of renal function or failure [25]. Cystatins belong to a group of proteins possessing a characteristic tertiary structure and a capacity to tightly, but reversibly, bind the active site of cysteine proteases (cathepsins) and thus inhibit their activity. As a renal marker, cystatin C (CysC) is an endogenous kidney function marker independent from non-renal markers such as gender, age, diet, and muscle mass. Since its levels are almost only dependent on kidney function, CysC is a very useful marker for kidney damage and GFR. [26].

For adult females, the creatinine level reference range is 44–97 μmol/L. The reference range for urea in blood or serum is 1.8–7.1 mmol/L, and for CysC, it is 0.57–1.79 μg/mL. In pregnant women, the reference ranges are in the lower ranges of the non-pregnant reference levels, with the lowest levels in the third trimester [27].

This study seeks to fill this gap in the research literature in pregnant women from the Republic of Suriname, an upper-middle income country. Suriname is situated on the northeastern coast of South America. Ninety percent of the population (estimated at 590,549 people) live in the capital Paramaribo and along the coastal area. The remainder live in the tropical rainforest interior that covers 90% of the country’s landmass. Suriname’s multi-ethnic population consists of five main groups: Hindustani (27%), Tribal (formerly Maroons, 22%), Creole (16%), Javanese (14%), and Indigenous (formerly Amerindians, 4%) [28].

The Caribbean Consortium for Research in Environmental and Occupational Health (CCREOH) is a prospective environmental epidemiological cohort study and has enrolled 1,189 mother/child dyads from urban, suburban, and interior areas of Suriname [29]. The study assesses exposure to toxic metals (e.g., lead and mercury) and other chemicals in pregnant women and investigates associations with maternal health, birth outcomes, and children’s neurodevelopment. Research thus far has found exposure to levels of mercury and lead that are of concern, especially in women living in the interior of Suriname. Mercury levels in hair ranged from below the limit of detection (0.05 μg/g) up to 31.5 μg/g and in blood from 0.51 to 47.9 μg/L, which are more than 10 times higher than the recommended or suggested public health action levels for mercury (1.1 μg/g in hair and 3.5 μg/L in blood) [30]. Preliminary CCREOH results indicate that lead levels are quite elevated as well. Since the WHO has stated that there is no known level of lead exposure that is considered safe [31], these findings are reason for concern.

Because of the magnitude of exposure in some of the CCREOH women, we assessed the adverse influences of mercury and lead on their liver and kidney function. Specifically, we determined mercury and lead levels in blood and examined the association with biomarkers of kidney and liver function in a sub cohort of these women. We also assessed differences in these functions between women with different exposure levels and demographics. 

## 2. Materials and Methods

### 2.1. Study Population

**Figure 1:**
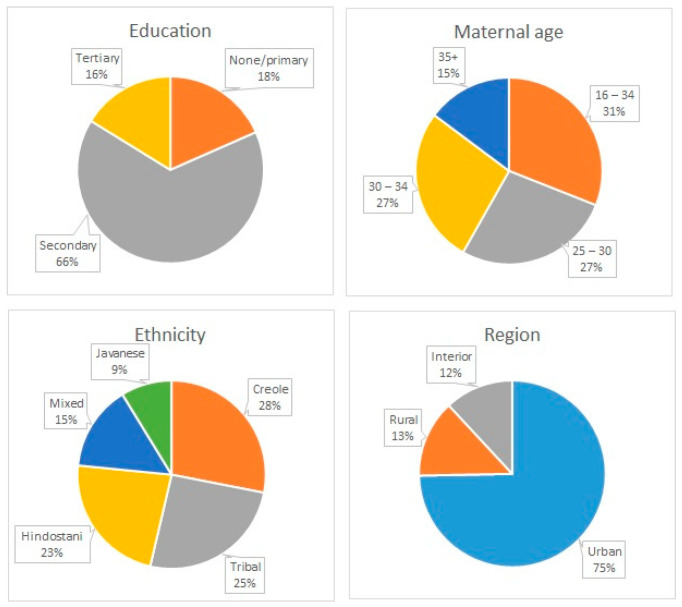
Maternal demographic data.

From December 2016 to July 2019, 1189 pregnant women between 16 and 45 years of age were recruited for the CCREOH study from three regions in Suriname: the capital city of Paramaribo (urban), the coastal and agriculturally rich district of Nickerie (rural), and the interior, where there are active ASGM activities and consumption of manioc and game is common [16]. Women were eligible to participate if they were 16 years of age or older; spoke Dutch, Saramaccan, or Trio; had a singleton gestation; and were planning to give birth at one of the study sites. The CCREOH study has been described in detail by Zijlmans et al. [29]. Maternal demographics are presented in Figure 1 and Table 1. Most pregnant women identified as Creole (103, 26.0%) followed by Tribal (94, 23.7%), Hindustani (84, 21.2%), Mixed (54, 13.6%), Javanese (32, 8.1%), or Indigenous (29, 7.3%). The majority had a secondary education (259, 65.4%) followed by none or primary (73, 18.4%) or tertiary (64, 16.2%). Maternal age at intake ranged from 16 to 40+ years, with 124 (30.4%) between 16 and 25 years old. In total, 296 (74.7%) were from the urban area, 53 (13.4%) were from rural areas, and 47 (11.9 %) were from the interior.

Blood collection, carried out through standard venipuncture into sterile vacutainers by trained health professionals, was conducted at two time points (late first/early second and third trimester) during pregnancy. We measured heavy metal concentrations in blood samples collected during the late first trimester/early second trimester in a subset of 400 women selected from the larger cohort. To further clarify, this project examined exposure to heavy metals early in the study prior to the recruitment and enrollment of the full cohort reported above or elsewhere. To date, exposure to heavy metals has not been determined in the remainder of the full cohort. Therefore, the participants examined in this project represent the subjects recruited and enrolled in the early part of the larger study. Other than being participants enrolled early in the study, no other selection criteria were used in selecting these samples and lead. In total, 341 serum samples were available to be tested for liver and kidney function. For those participants for whom we did not have a corresponding serum sample (i.e., those missing kidney and liver biomarker data), there was no evident bias with respect to the larger dataset in terms of ethnicity, household income, age at delivery, or education. This was determined through Chi-square analysis using *p* < 0.05 as the threshold for statistical significance (data not shown). 

This study was approved by the Institutional Review Board (IRB) of Tulane University and the Medical Ethical Commission of Suriname’s Ministry of Health (VG 023-14). All of the included women (18+) provided written informed consent, and assent was obtained from women who were 16 or 17 years old.

### 2.2. Liver and Kidney Function Assessments

Liver and kidney function were analyzed using internationally recognized analytical methods on the DXC-600 Beckman Coulter (Synchron systems). Urea, AST, and GGT were measured by enzymatic rate methods [32,33,34]. ALT was measured by a kinetic rate method. Creatinine concentration was assessed using the modified-rate Jaffe method. In this reaction, creatinine combines with picrate in an alkaline solution to form a creatinine–picrate complex [35]. Cystatin C is a particle-enhanced turbidimetric immunoassay (PETIA) [36].

### 2.3. Collection and Heavy Metal Analysis

Whole blood was collected in trace element vacutainers by venipuncture during pregnancy (late first/early second trimester). Whole blood samples were processed and stored frozen at −80 °C in the Clinical Chemistry Laboratory of the Academic Hospital in Paramaribo, Suriname. Samples from 400 participants were shipped frozen on dry ice to the Wisconsin State Laboratory of Hygiene Trace Element Research Laboratory (Madison, WI, USA) using a formal chain-of-custody process. Concentrations of lead and mercury were determined using Magnetic Sector Inductively Coupled Plasma Mass Spectrometry Formatting… Standard reference materials, matrix spikes, and method duplicates were used for quality assurance and quality checking (QA/QC). All internal and external QA/QC were acceptable.

### 2.4. Data and Statistical Analysis

Distributions of kidney and liver function biomarkers (Creat, urea, CysC, AST, ALT, and GGT) as well as lead and mercury in blood were tested for normality using the Shapiro–Wilk test, while homogeneity of variances was tested using the Levene test. The assumption of normality and homogeneity of variances were not met for any of the above-mentioned variables. The variables were therefore presented using medians and interquartile ranges. Bivariate associations between the outcome variables and demographic variables were tested using the Kruskal–Wallis test, while pairwise comparisons were conducted using the Dunn test. The Bonferroni correction was used to adjust for type 1 error inflation resulting from multiple comparisons on the same data. Kidney and liver function biomarkers were log transformed, and weighted least squares regression analysis was used to develop a predictive model for the outcome variables. All analyses were conducted using SPSS version 27 at the 5 percent level of significance. 

## 3. Results

### 3.1. Blood Level Values for Liver and Kidney Function Biomarkers

Mercury and lead levels in blood ranged from 0.18 to 39.18 μg/L and from 0.39 to 33.88 μg/dL, respectively (Table 2). Liver function biomarker ranges were as follows: ASAT 3-68 U/L, ALAT 4-77 U/L, and GGT 1-47 U/L (Table 2). Kidney function biomarker ranges were as follows: urea: 0.9–7.7 mmol/L, creatinine: 27–113 µmol/L, and CysC: 0.0–1.46 mg/L (Table 2).

### 3.2. Kidney Function

Significantly higher creatinine values were observed among participants with tertiary education and higher education compared to those with a secondary education (KW = 7.69, *p* = 0.021). Participants of Creole descent had higher creatinine levels than those from Hindustani, Indigenous, or Mixed descent (KW = 33.53, *p* < 0.001). Participants living in urban areas had higher creatinine levels than participants living in rural areas (KW = 23.61, *p* < 0.001). Participants aged 30–34 years had higher creatinine levels than those aged 16–24 years (KW = 8.97, *p* = 0.03). Creatinine levels increased with age, going from 47.39 U/L in women aged 16–19 years to 52.08 U/L in women aged 35 years and older. Although urea levels were initially statistically significantly different with respect to ethnicity (KW = 12.68, *p* = 0.027) and age (KW = 8.19, *p* = 0.043), differences among ethnic groups were not significant after adjusting for multiple comparisons.

Women in the age group of 35+ years had significantly higher CysC values than younger women aged 16–24 years (KW = 12.63, *p* = 0.006).

### 3.3. Liver Function

Region and ethnicity were significantly associated with liver function (Table 3). Rural participants had significantly higher levels of AST (KW = 17.94, *p* < 0.001) and ALT (KW = 9.98, *p* = 0.007) and significantly lower levels of GGT (LW = 14.41, *p* < 0.001) than urban and interior participants. Interior participants had significantly higher AST values than urban participants (KW = 17.94, *p* < 0.028). Indigenous participants had significantly higher ALT levels than Tribal participants (KW = 17.50, *p* = 0.004). Hindustani participants had significantly lower levels of GGT than Tribal, Mixed, or Creole participants (KW = 35.64, *p* < 0.001).

### 3.4. Heavy Metals

Heavy metal concentrations differed with respect to all sociodemographic variables, including education, region, ethnicity, and age (only mercury for age; Table 3 and Table 4). 

### 3.5. Weighted Least Squares Regression Predictive Modeling

The results for the predictive model for regression analysis are presented in Table 5. For all but AST and GGT, there were one or more significant predictors. 

The weighted least squares analysis adjusted for all variables showed significant predictors for creatinine (age, region at intake, and blood mercury levels). Age, district at intake, and blood mercury and lead levels were significant predictors for urea levels, while age was a significant predictor for CysC levels. For liver function, age and blood lead levels were significant predictors for ALT. The other liver function markers had no significant predictors.

## 4. Discussion 

In this study, we found a significant positive association between blood mercury levels and the kidney biomarkers creatinine and urea. No associations were found between blood mercury and markers of liver function. Blood lead levels and urea were negatively associated, while other kidney markers showed no significant associations. Blood lead levels were negatively associated with serum ALT and AST although only statistically significant for ALT. The blood mercury and lead levels were higher in pregnant women from the interior compared to in the women from urban and rural areas, in women with lower education, and in women identifying as Indigenous or Tribal. Blood mercury levels tended to increase with age.

### 4.1. Kidney Function

All kidney function test results were within the accepted reference ranges for healthy humans, with the exception of cystatin C, in which 94.8% of the results were within the reference range. Reference ranges for pregnant women are within the reference range for non-pregnant women. Any deviation from normal was therefore not noticed. Creatinine and urea are not likely to detect early or moderate loss of kidney function because these parameters typically increase when more than half of kidney function is lost [37]. 

### 4.2. Liver Function

Liver function test results were mostly within the reference range. For ALT and AST, two women (0.6%) had measured levels higher than the reference value. For GGT, 7 women (7%) had measured levels higher than the reference value. Reference ranges for pregnant women are within the reference ranges for non-pregnant women [27]. 

### 4.3. Mercury

#### 4.3.1. Kidney Function

In this study, we found a significant positive association between blood mercury levels and creatinine and urea, however as noted above, both biomarkers were within the normal, healthy reference ranges for pregnant women. A review study found that the effects of mercury on renal function were common [8]. This was demonstrated by increased excretion of N-acetyl-β-d-glucoaminidase (NAG), a biomarker of damage to the proximal tubules and clinical diagnosis of kidney dysfunction [8]. In a study on miners in the ASGM districts of Colombia, no effect of mercury on kidney function was found when using creatinine, albumin, and excretion of β-2 microglobulin as renal markers [38]. Previous research indicates that much of the mercury in these pregnant women is methylmercury, which is not typically considered to be as toxic to the kidney as elemental and inorganic forms of mercury [30,39]. As our study may indicate that there is some small influence or effect of mercury on kidney function in these pregnant women, it is possible that pregnant women may be more sensitive to mercury, including methylmercury, as well as to the smaller fractions of inorganic mercury comprising their total body burden. 

#### 4.3.2. Liver Function

In this study, we found no significant associations between blood mercury levels and liver function tests. Other studies mentioned below, however found different results. In a US study that included 3,769 adults (49.4% male and 50.6% female aged 20 years and older) that compared liver function and mercury levels in blood and urine, it was demonstrated that elevated liver enzymes were correlated with decreasing urinary mercury values. It was suggested that impaired liver function may lead to reduced demethylation, resulting in an increased level of organic mercury as a fraction of total blood mercury. Elevation of enzymes is therefore associated with decreased elimination of mercury in urine. 

#### 4.3.3. Demographics

The significantly elevated age-related blood mercury levels for women 30 years and older vs. younger women are consistent with the findings from a Korean study that illustrated higher blood lead and mercury levels in an mixed adult population older than 40 years of age [40]. In our study, blood mercury levels are the lowest in the age category 16–24 years and highest in women aged 30 years and older, with a peak of 3.48 µg/L for the category between 30 and 34 years. This age-associated increase in blood mercury levels is also reported for pregnant women in the US National Health and Nutrition Examination Study (NHANES), where geometric mean mercury levels increased with age from 0.56 µg/L for women aged 18–24 years to 1.01 µg/L for women aged 35 years and over [41]. Women in the interior had the highest blood mercury levels (9.70 μg/L). This may be explained by the fact that people in the interior depend on fish for their daily protein intake. Fish contaminated with mercury in the interior of Suriname has been previously described in place where there are ongoing mercury-based ASGM activities [39,42,43]. 

### 4.4. Lead 

#### 4.4.1. Kidney Function

Blood lead levels and urea exhibited a negative association, while other kidney biomarkers showed no significant associations, suggesting little effect on kidney function in these pregnant women at these lead concentrations. A meta-analysis by Kuraeiad et al. showed that high blood lead levels had an overall negative effect on kidney functions [44]. However, some individual studies, such as the one by Yao et al., who studied a Chinese population of men and women aged 20 years and older living in a region with heavy metal pollution, did not show a relation between blood lead levels and kidney function [45]. Another study found impacts on the kidney function parameters creatinine and urea in adults exposed to high concentrations of lead, especially concentrations >10 μg/dL [46]. Our study suggests that lead exposure may have a small effect on kidney function in these pregnant women; however, that effect is difficult to reconcile, as the association appears to be negative. This contrasts with other studies examining the effects of lead on kidney function in adults. This may be explained by an unknown confounder influencing this apparent relationship.

#### 4.4.2. Liver Function

We found a negative association between blood lead and ALT levels, while there were no other significant associations between liver function biomarkers and lead. This apparent negative association is difficult to reconcile with both our expectations of liver toxicity and the current evidence regarding liver function and lead. We did not find any other studies that describe BLL and liver function in pregnant women. However, other studies found significant positive correlations with lead exposure and liver function in adult non-pregnant women between 33 and 79 years. Lee et al. found that BLLs were significantly correlated with GGT (r = 0.133, *p* = 0.015), AST (r = 0.172, *p* = 0.002), and ALT (r = 0.171, *p* = 0.002) [20]. In studies that report significant positive correlations between BLL and liver function, participants have higher blood lead levels compared to our population, suggesting that liver function is likely compromised at higher lead exposure levels. Perhaps our findings reflect the influence of an unmeasured confounder that is possibly related to lead or that is simply correlated with lead in this case. We also note here that ALT alone is usually not a good indicator of liver damage or impaired liver function

#### 4.4.3. Demographics

Most (88.6%) of the blood lead levels (BLL) in our pregnant women were higher than 0.92 µg/dL, the average BLL measured in 2015-2016 in American adults aged 20 years and older [47]. In addition, 15.2% of these women were above the US Centers for Disease Control blood lead reference value of 5 µg/dL. In the NHANES between 2003 and 2008, the geometric mean BLL for pregnant women was 0.64 μg/dL [41], and in the EDAN cohort study, the mean levels varied between 1.9 and 2.2 µg/dL [48].

In a study on pregnant Bangladeshi women, the mean BLL was 4.7 μg/dL (SD 3.6%), which is higher than the mean level we found in our study participants (2.99 µg/dL). Almost one-third (31%) of the Bangladeshi participants had BLLs higher than 5 µg/dL, with 6% having levels exceeding 10 µg/dL [49]. Women in the interior had the highest blood lead levels (5.80 μg/L). This may be attributed to the consumption of wild game harvested with lead shots [16] as well as manioc consumption, as manioc is prepared using large metal pans that may contain lead, as is the case in other Amazonian regions [16,50,51].

### 4.5. Kidney Function Demographics

#### 4.5.1. Age

Both creatinine and cystatin C significantly increased with age, indicating a decline in kidney function with age. This compares to findings that aging is associated with changes in kidney morphology, leading to the decline of kidney function [52]. Kidney decline with age is described in two studies in which the decline in GFR is reported to be between −7.5 mL/min and 6.3 mL/min per decade [52,53]. A Japanese study in pregnant and non-pregnant women measured kidney function during the various stages of pregnancy. Lin et al. showed a decline in kidney function of 25% caused by lower physical function in middle-aged and elderly women [54]. Our statistically weighted model adjusted for age showed that creatinine remained significant.

#### 4.5.2. Geographic Region

District at intake was negatively associated with creatinine in women from urban areas, who had higher creatinine values than women from rural areas. Since this was the only association that was found in our study, it may suggest subtle effects on renal function attributable to regional differences. Assessments of risk factors of kidney impairment such as diabetes, obesity, and hypertension were not included in our study. However, they may contribute to the geographical differences we found in kidney function impairment.

#### 4.5.3. Ethnicity and Socioeconomic Status

Kidney disease differentially affects ethnic groups. An American study showed that African Americans, Native Americans, Asians, and Hispanics in the US have a double to four-fold higher risk of end-stage renal disease (ESRD). The same paper mentioned that access to care and healthcare professionals is one of the possible causes for this [55]. In our study, we found that women with lower education levels had significantly higher blood mercury and lead levels. In a review, Hossain et al. found that people with lower SES (educational and/or occupational and/or income) had a higher risk of CKD. Access and adherence to healthcare were mentioned as possible reasons [56]. We found higher blood creatinine levels in women of Creole descent. However, this cannot be attributed to exposure to mercury or lead because they had lower exposure levels than the other groups. Similar results were found in the third National Health and Nutrition Examination Survey (NHANES III), which measured serum creatinine levels in the US population. The values for creatinine were higher in the non-Hispanic Black population [57]. Physiological differences such as muscle mass and non-communicable diseases (NCDs) such as hypertension or diabetes may be possible explanations for the differences. The ethnic differences in creatinine are not related to mercury or lead exposure.

### 4.6. Liver Function Demographics

#### 4.6.1. Geographic Location

In our study, there was a significant difference in the liver function levels between the rural and urban and interior participants. ALT levels in rural participants were higher than those in the other two regions, whereas GGT levels were lower. 

#### 4.6.2. Socioeconomics

The GGT levels for Hindustani participants were significantly lower than they were Tribal, Mixed, or Creole participants. The AST levels for Indigenous participants were significantly higher than the levels for Tribal participants.

We could not find any comparative study for these findings

### 4.7. Limitations

Confounding factors that can influence kidney or liver function, such as use of medication or diet during pregnancy, were not systematically determined in this study. Other causes for higher mercury and lead levels include the presence of metabolic syndrome, which we did not include in this study. Studies in adolescents and adults found higher levels of serum mercury and lead levels in those with metabolic syndrome compared to in those without [18,58]. The decision to select 400 samples at the beginning of the study may result in the 400 samples not being representative for the entire cohort.

## 5. Conclusions 

This study examined the relationship between blood levels of mercury and lead and possible relationships with liver and kidney function in pregnant women from the CCREOH cohort living in Suriname. We found concerningly high blood levels of both mercury and lead in these women. Specifically, women living in the interior of Suriname had higher blood mercury and lead levels than women living in urban and rural areas. In addition, our findings suggest a relationship between high mercury blood levels and potential harmful effects on liver and kidney function. Both creatinine and urea were elevated with higher mercury blood levels. ALT levels were lower with elevated BLL, which, as noted above, is counterintuitive to our current understanding of the biological and toxicological effects of lead. This could be due to a possible mitigating effect of genetic or nutritional factors. 

## Figures and Tables

**Table 1:** Concentrations of biomarkers of liver and kidney function are presented, as are concentrations of lead and mercury in blood samples from the participants in various categories. Data are presented as medians with interquartile ranges (IQR).

Variable	N (%)	Bl Cr		Bl Ur		CysC		AST		ALT		GGT		Bl Hg (µg/L)		Bl Pb (µg/dL)	
		Median	IQR	Median	IQR	Median	IQR	Median	IQR	Median	IQR	Median	IQR	Median	IQR	Median	IQR
Age	N (%)																
16–24 years	124 (30.4)	47	11	2.3	0.7	0.6	0.2	12	6	9	5	10	8	2.39	2.64	1.92	1.56
25–29 years	109 (26.7)	47.5	14	2.5	0.8	0.61	0.16	12	4	10	3	9.5	8	2.46	2.81	2.01	1.71
30–34 years	108 (26.5)	51	15	2.5	0.8	0.65	0.14	11	4	9	4	11	8	3.48	3.66	2.19	3.48
35+ years	59 (14.5)	50	15	2.7	0.9	0.67	0.2	11	5	9	4	12	9	3.31	2.72	2.14	1.65
Ethnicity	N (%)																
Creole	103 (26)	53	13	2.4	0.9	0.605	0.17	11.5	4	9	4	12.5	8	2.39	1.93	1.86	1.73
Hindustani	84 (21.2)	43	14	2.6	0.6	0.63	0.18	11	5	9	3	8	6	2.97	2.22	1.75	1.17
Indigenous	29 (7.3)	43	9	2.3	0.6	0.6	0.78	14	8	12	7	9	11	7.92	20.23	5.65	4.77
Javanese	32 (8.1)	51	11	2.6	0.6	0.68	0.15	11	6	10	3	10	6	3.44	2.61	1.98	1.54
Tribal	94 (23.7)	49	11	2.3	0.9	0.6	0.2	12	4	8	4	11	11	4.25	8.96	2.94	3.87
Mixed	54 (13.6)	47	13	2.5	0.8	0.63	0.14	11	5	9	3	11	7	2.25	2.57	1.62	1.2
Region																	
Urban	296 (74.7)	50	14	2.5	0.8	0.62	0.19	11	4	9	4	11	8	2.55	2.34	1.81	1.34
Rural	53 (13.4)	42	8	2.6	0.5	0.64	0.13	14	7	11	5	7	7	2.63	2.64	2.01	1.75
Interior	47 (11.9)	46	9	2.3	0.7	0.6	0.18	12	4	9	4	11.5	11	9.7	8.92	5.08	6.13
Education																	
None or primary	73 (18.4)	47	12	2.4	0.7	0.615	0.17	12	4	9	4	9	12	4.41	10.85	3.97	5.79
Secondary	259 (65.4)	47	12	2.5	0.8	0.61	0.22	11	5	9	4	11	9	3.44	3.42	2.19	1.46
Tertiary	64 (16.2)	50	13	2.5	0.7	0.63	0.14	11	5	9	3	10	7	2.19	2	1.51	1.1

Bl Cr (blood creatinine); Bl Ur (blood urea); CysC (cystatin C); AST (aspartate amino transferase); ALT (alanine amino transferase); GGT (gamma-glutamyl transferase); Bl Hg (blood mercury); Bl Pb (blood lead).

**Table 2:** Distribution of mercury and lead concentrations and biomarkers for liver and kidney function.

	Valid					
	N	Missing	Mean	Median	Min	Max
Mercury (μg/L)	400	8	4.42	2.96	0.18	39.18
Lead (μg/dL)	400	8	2.99	1.99	0.39	33.88
Creat (μmol/L	341	67	49.40	49.00	27	113
Ureum (mmol/L)	341	67	2.539	2.500	0.9	7.7
CysC (μg/L)	332	76	0.5945	0.6200	0.00	1.46
ASAT (U/L)	340	68	12.42	11.00	3	68
ALAT (U/L)	340	68	10.15	9.00	4	77
GGT (U/L)	340	68	12.70	10.00	1	47

**Table 3:** Results of examining maternal demographic characteristics and biomarkers of effect (liver and kidney function) and exposure (mercury and lead in blood).

	Variable	KW ^a^	*p*-Value	KW	*p*-Value	KW	*p*-Value
		Creatinine		Urea		CysC	
Kidney function	Education	7.69	0.021	0.60	0.745	0.90	0.638
	Ethnicity	33.52	<0.001	12.68	0.027	6.00	0.306
	Region	23.61	<0.001	3.92	0.141	1.62	0.446
	Age	8.97	0.03	8.19	0.042	12.63	0.006
		AST		ALT		GGT	
Liver function	Education	2.45	0.293	3.74	0.154	2.60	0.273
	Ethnicity	6.99	0.221	17.50	0.004	35.64	<0.001
	Region	17.94	<0.001	9.98	0.007	14.41	<0.001
	Age	4.02	0.259	4.56	0.207	4.01	0.261
		Hg		Pb			
Heavy metals	Education	45.36	<0.001	72.80	<0.001		
	Ethnicity	57.80	<0.001	72.35	<0.001		
	Region	72.71	<0.001	75.15	<0.001		
	Age	18.95	<0.001	1.10	0.78		

^a^ KW: Kruskal–Wallis test statistic.

**Table 4:** Results of examining the relationships between categories within maternal demographic characteristics and exposure to lead and mercury. Median values are reported in Table 1.

Variable	Dunn Pairwise	Adjusted *p*-Value	
		Hg	Pb
Education	Tertiary–Secondary	0.017	0.019
	Tertiary–None or Primary	0	0
	Secondary–None or Primary	0	0
Ethnicity	Mixed–Tribal	0	0
	Mixed–Indigenous	0.0007	0
	Hindustani–Tribal	0	0
	Hindustani–Indigenous	0.028	0.001
	Javanese–Tribal	-	0.01
	Javanese (1.98)–Indigenous	-	0.029
	Creole–Tribal	0	0
	Creole–Indigenous	0.003	0.012
Region	Rural (2.63)–Urban (2.55)	1	1
	Rural (2.63)–Interior (9.70)	0	0
	Urban (2.55)–Interior (9.70)	0	0
Maternal age	16–24 years–30–34 years	0.003	-
	16–24 years–35+ years	0.002	-

**Table 5:** Results from the weighted least squares regression analysis examining the relationship between maternal demographic characteristics and heavy metal exposure with the liver and kidney function biomarkers.

		Unstandardized Coefficients	95.0% Confidence Interval for B	
		B	Lower Bound	Upper Bound
Bl Cr	maternal age at delivery	1.382 *	0.386	2.377
	district at intake	−3.577 *	−5.157	−1.998
	ethnicity	−0.419	−0.98	0.142
	education	0.178	−1.89	2.246
	blood mercury level	0.311 *	0.046	0.576
	blood lead level	0.07	−0.309	0.449
Bl Ur	maternal age at delivery	0.102 *	0.033	0.172
	district at intake	−0.11 *	−0.213	−0.006
	ethnicity	−0.02	−0.056	0.017
	education	−0.082	−0.222	0.057
	blood mercury level	0.03 *	0.014	0.046
	blood lead level	−0.042 *	−0.075	−0.008
CysC	maternal age at delivery	0.04 *	0.018	0.063
	district at intake	−0.019	−0.068	0.03
	ethnicity	−0.003	−0.015	0.009
	education	0.017	−0.029	0.062
	blood mercury level	−0.002	−0.008	0.005
	blood lead level	0.003	−0.007	0.014
AST	maternal age at delivery	−0.429	−1.012	0.153
	district at intake	0.981	−0.174	2.136
	ethnicity	−0.029	−0.341	0.283
	education	0.266	−0.891	1.422
	blood mercury level	0.118	−0.057	0.293
	blood lead level	−0.123	−0.332	0.086
ALT	maternal age at delivery	−0.483 *	−0.943	−0.022
	district at intake	0.393	−0.432	1.217
	ethnicity	−0.046	−0.301	0.208
	education	0.347	−0.438	1.131
	blood mercury level	0.171	−0.005	0.348
	blood lead level	−0.136 *	−0.185	−0.087
GGT	maternal age at delivery	0.037	−0.751	0.825
	district at intake	−1.155	−2.791	0.481
	ethnicity	0.192	−0.213	0.598
	education	−0.594	−2.106	0.918
	blood mercury level	0.158	−0.092	0.408
	blood lead level	0.007	−0.143	0.158

Bl Cr (blood creatinine); Bl Ur (blood urea); CysC (cystatin C); AST (aspartate amino transferase); ALT (alanine amino transferase); GGT (gamma-glutamyl transferase). * indicates a significant association *p* < 0.05.

## Data Availability

Data are available upon reasonable request.
